# Supplementary material for: Health-Related Quality of Life in Patients with Chronic Myeloid Leukemia Treated with First- Versus Second-Generation Tyrosine Kinase Inhibitors
Source: J Clin Med. 2020 Oct 25;9(11):3417. doi: 10.3390/jcm9113417 (PMC7692789; doi:10.3390/jcm9113417)
Supplement: Supplementary file 1 [file jcm-09-03417-s001.pdf]

Journal of
Clinical Medicine

MDPI

**Supplementary data 1**

Code ________

<u>Opening page for the research</u>

Greetings,

This research study was initiated at the Davidoff Cancer Center, Rabin Medical Center, in cooperation with the CML patient organization with the purpose to learn from you, our patients, how the disease and treatment affects your quality of life. We assume most or all of you are treated with one of the customary medications: Imatinib, Dasatinib, Nilotinib, Bosutinib and Ponatinib.

In the questionnaire before you, are questions regarding the disease, the treatment's side effects, the physical and emotional coping with this state and finally, demographic questions. If you do not feel comfortable answering certain questions, please feel free to skip any of them. This questionnaire should take you approximately 15 minutes to complete.

Responding to this questionnaire provides us with the necessary consent by you to participate in this research project.

If you have stopped treatment, please answer only the relevant questions and state that you have stopped treatment.

The data will be coded and kept by the principal investigators, Dr. Adi Shacham and Dr. Uri Rozovski until the study is completed.

In addition to answering the questionnaire, if the medical center in which you are treated will consent to participate in the study and if the study is approved by your institution review board, we will collect more data from your medical record (demographic details, CML disease data, comorbidities, the course of the disease (CML) and the treatments you received, side effects, response to treatment and laboratory data).

The data will be collected by a research coordinator who will be appointed by our research team (Davidoff Cancer Center at Rabin Medical Center and the CML patient organization). Collection and maintenance of the data will be encrypted (coded) and will be kept by the principal investigators, Dr. Adi Shacham and Dr. Uri Rozovski until the study is completed.

If the medical center you are caring for will not consent to the study, we can only use data from the questionnaires. In any case, at any stage of filling out the questionnaire or participating in the study, you can stop or quit your participation in the research.

In any research related matter you can contact Dr. Adi Shacham, at 03-9378008/9, hematological institute secretary.

Thank you very much for participating in the study!

- Dr. Adi Shacham, Hematology, Rabin Medical Center

- Dr. Uri Rozovski, Hematology, Rabin Medical Center

- Giora Sherf

**EORTC QLQ-C30 version**(3)

We are interested in some things about you and your health. Please answer all of the questions yourself by circling the number that best applies to you. There are no "right" or "wrong" answers. The information that you provide will remain strictly confidential

Please fill in your initials:
Your birthdate (Day, Month, Year):
Today's date (Day, Month, Year):

| | Not at All | A Little | Quite a Bit | Very Much |
|---|---|---|---|---|
| 1. Do you have any trouble doing strenuous activities, like carrying a heavy shopping bag or a suitcase? | 1 | 2 | 3 | 4 |
| 2. Do you have any trouble taking a long walk? | 1 | 2 | 3 | 4 |
| 3. Do you have any trouble taking a short walk outside of the house? | 1 | 2 | 3 | 4 |
| 4. Do you need to stay in bed or a chair during the day? | 1 | 2 | 3 | 4 |
| 5. Do you need help with eating, dressing, washing yourself or using the toilet? | 1 | 2 | 3 | 4 |

**During the past week:**

| | Not at All | A Little | Quite a Bit | Very Much |
|---|---|---|---|---|
| 6. Were you limited in doing either your work or other daily activities? | 1 | 2 | 3 | 4 |
| 7. Were you limited in pursuing your hobbies or other leisure time activities? | 1 | 2 | 3 | 4 |
| 8. Were you short of breath? | 1 | 2 | 3 | 4 |
| 9. Have you had pain? | 1 | 2 | 3 | 4 |
| 10. Did you need to rest? | 1 | 2 | 3 | 4 |
| 11. Have you had trouble sleeping? | 1 | 2 | 3 | 4 |
| 12. Have you felt weak? | 1 | 2 | 3 | 4 |
| 13. Have you lacked appetite? | 1 | 2 | 3 | 4 |
| 14. Have you felt nauseated? | 1 | 2 | 3 | 4 |
| 15. Have you vomited? | 1 | 2 | 3 | 4 |
| 16. Have you been constipated? | 1 | 2 | 3 | 4 |

**During the past week:**

| | Not at All | A Little | Quite a Bit | Very Much |
|---|---|---|---|---|
| 17. Have you had diarrhea? | 1 | 2 | 3 | 4 |
| 18. Were you tired? | 1 | 2 | 3 | 4 |
| 19. Did pain interfere with your daily activities? | 1 | 2 | 3 | 4 |
| 20. Have you had difficulty in concentrating on things, like reading a newspaper or watching television? | 1 | 2 | 3 | 4 |
| 21. Did you feel tense? | 1 | 2 | 3 | 4 |
| 22. Did you worry? | 1 | 2 | 3 | 4 |
| 23. Did you feel irritable? | 1 | 2 | 3 | 4 |
| 24. Did you feel depressed? | 1 | 2 | 3 | 4 |
| 25. Have you had difficulty remembering things? | 1 | 2 | 3 | 4 |
| 26. Has your physical condition or medical treatment interfered with your <u>family</u> life? | 1 | 2 | 3 | 4 |
| 27. Has your physical condition or medical treatment interfered with your <u>social</u> activities? | 1 | 2 | 3 | 4 |
| 28. Has your physical condition or medical treatment caused you financial difficulties? | 1 | 2 | 3 | 4 |

**For the following questions please circle the number between 1 and 7 that best applies to you**

29. How would you rate your overall <u>health</u> during the past week?

| 1 | 2 | 3 | 4 | 5 | 6 | 7 |
|---|---|---|---|---|---|---|
| Very poor | | | | | | Excellent |

30. How would you rate your <u>overall quality of life</u> during the past week?

| 1 | 2 | 3 | 4 | 5 | 6 | 7 |
|---|---|---|---|---|---|---|
| Very poor | | | | | | Excellent |

© Copyright 1995 EORTC Quality of Life Group. All rights reserved.
Version 3.

**EORTC QLQ-CML24**

Patients sometimes report that they have the following symptoms or problems. Please indicate the extent to which you have experienced these symptoms or problems during the past week. Please answer by circling the number that best applies to you.

—

**During the past week:**

| | Not at All | A Little | Quite a Bit | Very Much |
|---|---|---|---|---|
| 31. Have you had abdominal pains or cramps? | 1 | 2 | 3 | 4 |
| 32. Have you had a dry mouth? | 1 | 2 | 3 | 4 |
| 33. Have you been concerned about changes in your weight? | 1 | 2 | 3 | 4 |
| 34. Have you had skin problems (e.g. color changes, itchy, dry or flaking skin)? | 1 | 2 | 3 | 4 |
| 35. Have you had headaches? | 1 | 2 | 3 | 4 |
| 36. Have you had aches or pains in your muscles or joints? | 1 | 2 | 3 | 4 |
| 37. Have you had hair loss? | 1 | 2 | 3 | 4 |
| 38. Have you sweated? | 1 | 2 | 3 | 4 |
| 39. Have you had acid indigestion or heartburn? | 1 | 2 | 3 | 4 |
| 40. Have you felt drowsy? | 1 | 2 | 3 | 4 |
| 41. Have you experienced any swelling in certain parts of your body (e.g. ankles, legs or around your eyes)? | 1 | 2 | 3 | 4 |
| 42. Have you had to urinate frequently? | 1 | 2 | 3 | 4 |
| 43. Have you had problems with your eyes | 1 | 2 | 3 | 4 |

| | | | | |
|---|---|---|---|---|
| (e.g. burning, watery, irritated or dry)? | | | | |
| 44. Have you had muscle cramps? | 1 | 2 | 3 | 4 |

**During the past week:**

| | Not at All | A Little | Quite a Bit | Very Much |
|---|---|---|---|---|
| 45. Have you had emotional ups and downs? | 1 | 2 | 3 | 4 |
| 46. Have you worried about your future health? | 1 | 2 | 3 | 4 |
| 47. Have you had any difficulties carrying on with your usual activities because of getting tired easily? | 1 | 2 | 3 | 4 |
| 48. Have you worried about getting an infection? | 1 | 2 | 3 | 4 |
| 49. Have you felt dissatisfied with your body as result of the disease or treatment? | 1 | 2 | 3 | 4 |
| 50. How much has your treatment been a burden to you? | 1 | 2 | 3 | 4 |
| 51. Have you needed social support (e.g. family, friends or relatives) to undergo therapy or to cope with the disease? | 1 | 2 | 3 | 4 |
| 52. Have you felt satisfied with the care you have received? | 1 | 2 | 3 | 4 |
| 53. Have you felt satisfied with the information you have received (e.g. about the disease and its treatment)? | 1 | 2 | 3 | 4 |
| 54. Have you felt satisfied with the quality of your social life (including family and/ or friends)? | 1 | 2 | 3 | 4 |

© Copyright 2011 EORTC Quality of Life Group. All rights reserved. Phase IV

Have you been suffering from the following symptoms since you started your current drug treatment for CML?

| **Circle the answer that suits you the most** | **Not at All** | **A Little** | **Quite a Bit** | **Very Much** |
|---|---|---|---|---|

| | | | | |
|---|---|---|---|---|
| 1. Anxiety? | 1 | 2 | 3 | 4 |
| 2. Has your work performance been impaired? | 1 | 2 | 3 | 4 |
| 3. Has your function at home been impaired? | 1 | 2 | 3 | 4 |
| 4. Has your sexual function been impaired? | 1 | 2 | 3 | 4 |
| 5. Has the CML disease or its treatment affected family planning (for example - pregnancy timing or number of children)? | 1 | 2 | 3 | 4 |
| 6. Have you started follow up at a cardiovascular /vascular /respiratory /neurology clinic? | | Yes | No | |
| 7. Have you suffered from breathing difficulty due to fluid accumulation in your lung? | | Yes | No | |

8. How many times have you been hospitalized in the past six months? ___________

9. How many times have you had to cancel plans during the past month because of your illness or treatment? ________

10. Do you use medical cannabis? Yes No

11. How many days during the past week did you use cannabis? ___________

12. How many days during the past week have you used painkillers (except cannabis)? __________

13. In your opinion, how many times have you missed a dose of your drug for CML in the last month? _________

14. Have you missed one or more doses of the drug for CML in the past week? Yes No

15. How would you rate your overall health over the past three months?

| 1 | 2 | 3 | 4 | 5 | 6 | 7 |
|---|---|---|---|---|---|---|
| Very poor | | | | | | Excellent |

16. How would you rate your overall quality of life in the past three months?

| 1 | 2 | 3 | 4 | 5 | 6 | 7 |
|---|---|---|---|---|---|---|
| Very poor | | | | | | Excellent |

17. In relation to the previous question, how much of your quality of life would you attribute to the CML disease or its treatment? Not at all A Little Quite a Bit Very much

In what way has your life changed since the discovery of the disease? Open question

______________________________________________________________________________

______________________________________________________________________________

______________________________________________________________________________

We will be happy to receive comments about the questionnaire

We will be happy to receive more relevant information you would like to share

**Demographic details**

1. Date of birth ________________
2. Country of birth _____________
3. Gender Male Female
4. Family status Single Married Widower
5. Number of children __________
6. Degree of religiosity Secular Traditional Religious Orthodox
7. Elementary school education Yes Partial No
8. High school education Yes Partial No
9. Academic education First degree Second degree or more No
10. City of living ______________

**Detail regarding the CML disease**

1. Estimated date of diagnosis _______________
2. What drug are you currently treated with?

Imatinib (generic) Glivec (original) Dasatinib (Sprycel) Nilotinib (Tasigna) Bosutinib (Bosulif) Ponatinib (Iclusig)

3. When did you start receiving the drug? estimated date _______________

4. Are you on a break, i.e do not receive tyrosine kinase inhibitors? Yes No

5. If you are on a treatment break, how many months are you off treatment? ____________

6. As far as you know, have you reached a good response to the current treatment? Yes Partial No

7. Have you received any prior medication before the current treatment?

8. If so, what drugs? Check one or more of the options

| | | | | | |
|---|---|---|---|---|---|
| Imatinib (generic) | Glivec (original) | Dasatinib (Sprycel) | Nilotinib (Tasigna) | Bosutinib (Bosulif) | Ponatinib (Iclusig) |

9. As far as you know, why was the previous treatment been replaced?

   Side effects Resistance Inadequate response

10. Are you satisfied with the treatment you are currently receiving?

   Very Quite pleased Moderately satisfied Unsatisfied

**Identifying information**

First name ______________________________________________

Last name ______________________________________________

ID number ______________________________________________

Hospital ______________________________________________

Treating hematologist ______________________________________

Thank you very much for your cooperation
